# miR-148b Regulates Proliferation and Differentiation of Neural Stem Cells via Wnt/β-Catenin Signaling in Rat Ischemic Stroke Model

**DOI:** 10.3389/fncel.2017.00329

**Published:** 2017-10-20

**Authors:** Jingru Wang, Tuanzhi Chen, Guangzhen Shan

**Affiliations:** Department of Neurology, Liaocheng People's Hospital, Liaocheng, China

**Keywords:** ischemic stroke, microRNA-148b, Wnt/β-catenin signaling, neurogenesis, sub-ventricular zone

## Abstract

Stroke is the second leading cause of death worldwide. Stroke induced proliferation and differentiation of neural stem cells (NSCs) that have been proven to participate in ischemic brain repair. However, molecular mechanisms that regulate neurogenesis have not been fully investigated. MicroRNAs play an important role in the neurological repairing process and impact stroke recovery outcome. MiRNA-148b has been reported to regulate cell proliferation in tumor cells, but its role in NSCs after ischemic stroke remains unknown. Here, we found an overexpression of MiRNA-148b in subventricular zone (SVZ) of rat ischemic brain. In original cultured ischemic NSCs, transfection of MiRNA-148b mimic or inhibitor could suppress or enhance the expression of Wnt-1, β-catenin, and Cyclin D1, hence effected wnt/β-catenin signaling. MiRNA-148b inhibitor promoted NSCs proliferation and differentiation into newborn neural and astrocytes, and this action could be silenced with knockdown of Wnt-1. In middle cerebral artery occlusion (MCAo) rats, injection of MiRNA-148b inhibitor could reduce ischemic lesion volume and improve neurological function outcome. Collectively, our data suggest that MiRNA-148b suppressed wnt/β-catenin signaling attenuates proliferation and differentiation of neural stem cells, these findings shed new light on the role of MiRNA-148b in the recovery process during the stroke and contribute to the novel therapy strategy.

## Introduction

Stroke is the second leading cause of death worldwide (Donnan et al., 2008). Each year in the United States, 130,000 people die of stroke related diseases, and ~800,000 people suffer a stroke (Mozaffarian et al., 2015; Writing Group et al., 2016). In most Asian countries, stroke mortality was higher than western countries (Ueshima et al., 2008). More than 80% of stroke is ischemic stroke triggered by blood flow blockage within major cerebral arteries (Donnan et al., 2008). Until recently, the only FDA-approved treatment for acute ischemic stroke is intravenous recombinant tissue plasminogen activator (tPA), but only ~4.5% of the patients received the medicine since its narrow therapeutic window (Kleindorfer et al., 2013; Lackland et al., 2014).

Accumulated studies from experimental and clinical stroke showed that cerebral ischemia induces spontaneous brain repair processes, including neurogenesis, angiogenesis, oligodendrogenesis, and astrogliosis (Jin et al., 2006; Macas et al., 2006; Minger et al., 2007). Neurogenesis occurs mainly in two brain regions, the sub-ventricular zone (SVZ) of the lateral ventricles and the sub-granular zone (SGZ) in the hippocampal dentate gyrus (Alvarez-Buylla and Garcia-Verdugo, 2002; Zhao et al., 2008). Neural stem cells (NSCs) in SVZ divide into neural progenitor cells (NPCs) and oligodendrocyte progenitor cells (OPCs). In rodent ischemic stroke model, NSCs in SVZ generate a large amount of neuroblasts that migrate to ischemic boundary improving neurological functional recovery (Wang et al., 2012). However, these spontaneous brain repair processes are constrained with limited improvement outcome (Benowitz and Carmichael, 2010), the mechanism underlying of ischemic stroke and the recovery process need to be further exported in order to give rise the new therapeutic methods.

MicroRNAs (miRNA) are small non-coding RNAs of ~20 nucleotides that are crucial for many biological processes (Bartel, 2004). In the decade, more studies indicate miRNAs play an important role in the process of ischemic stroke (Kassis et al., 2017). Among these miRNAs, miRNAlet-7a, miRNA-124, and miRNA-137 were reported to induce neuroprotection after cerebral ischemia, while miRNA-34a, microRNA-181c, and miRNA-17–92 were reported to exacerbates brain injury in ischemic Stroke (Szulwach et al., 2010; Liu et al., 2013; Hamzei Taj et al., 2016; Liang and Lou, 2016; Ma et al., 2016; Wang et al., 2016).

MiRNA-148b belongs to miRNA-148/152 (miR-148/152) family, which has been elucidated to involved in various biological processes (Chen et al., 2013). miR-148b was reported to regulate the differentiation of mesenchymal stem cells in the process of early osteogenesis (Schoolmeesters et al., 2009), and involved in mouse adipogenesis via regulating PPARγ (John et al., 2012). Also, miR-148b suppressed hepatic cancer stem cell by targeting neuropilin-1 (Liu et al., 2015). All these results revealed miR-148b participate in cancer cell self-renewal and differentiation. Based on it, we infer miR-148b might affect the neurogenesis of NSCs and affect post-stroke recovery.

In our study, we first tested the impact of ischemia on miR-148b, then explored the regulation of miR-148b on NSCs neurogenesis and the mechanism beneath *in vitro*. We also tested the effect of miR-148b on neurological functional recovery *in vivo*. All the data implied that MiRNA-148b regulated proliferation and differentiation of NSCs in SVZ after ischemic stroke via Wnt/β-catenin signaling.

## Materials and methods

### Animal model

All experimental procedures were consistent with the Animal Use and Care of Medical Laboratory Animals from the Ministry of Public Health of People's Republic of China. Male adult Sprague-Dawley rats weighing 180–220 g were purchased from Animal Centre of Shandong University. Rats were subjected to middle cerebral artery occlusion (MCAo) as published protocol (Chen et al., 2001; Zhang L. et al., 2016). Briefly, rats were anesthetized with isoflurane (4% for induction and 2% for maintenance) in 70% N_2_O and 30% O_2_. Under the operating microscope, the right common carotid artery (CCA), external carotid artery (ECA), and internal carotid artery (ICA) were isolated. An 18-mm-long 4 ± 0 nylon filament with an expanded tip was gently advanced from the ECA into the lumen of the ICA. The tip of the filament was positioned at the origin of the middle cerebral artery (MCA).

### Dual-luciferase assay

Dual-Luciferase assay was performed follow by manufacture's instruction. Briefly, a segment of the 3′UTR of wild type Wnt1 gene (Wnt1-3′UTR) encompassing the miR-148b binding site was cloned in a pEZX-MT06 vector with Firefly/Renilla duo Luciferase reporter driven by a CMV promoter (Genecopoeia, USA). Point mutations of 3′UTR of Wnt1 gene (Wnt1-3′UTR-m) in miR-148b binding site were made as UGCACUG to UGCCGGG (Figure **2**). To test the interaction between miR-148b and 3′ UTR of Wnt1 gene, 293T cells (ATCC) were co-transfected with miR-148b mimics (100 ng/10^6^ cells) or miR-148b mimic negative control (miR-148b mimic-con, 100 ng/10^6^ cells) and Wnt1-3′UTR (100 ng/10^6^ cells) or Wnt1-3′UTR-m (100 ng/10^6^ cells) by using lipofectamine-2000 transfection reagent (Life technologies, USA). Twenty-four hours later, cells were lysed and treated with a Dual-luciferase assay kit (Genecopoeia, USA). Luciferase activity was detected using a multimode microplate reader (PerkinElmer/Fusion, USA) and normalized to its corresponding Renilla luciferase activity.

### Neural stem cells culture

Seven days after MCAo, NSCs were isolated from rats SVZ and cultured as previously published papers (Reynolds and Weiss, 1992; Morshead et al., 1994; Liu et al., 2016). NSCs were dissociated and expanded at a density of 2 × 10^4^ cells/ml in proliferation medium [DMEM/F-12 medium (Life Technologies), 20 ng/mL of EGF (R&D System), bFGF (R&D System)] in a cell culture incubator at 37°C, 5% CO_2_. NSCs differentiation were induced in differentiation medium [DMEM/F-12 medium contains L-glutamine (2 mmol/L, Life Technologies), glucose (0.6%, Sigma-Aldrich), NH_2_(CH_2_)_4_NH_2_ (9.6 mg/Ml, Sigma-Aldrich), insulin (0.025 mg/mL, Sigma-Aldrich), progesterone (6.3 ng/mL, Sigma-Aldrich), apo-transferrin (0.1 mg/mL, Sigma-Aldrich), and Na_2_SeO_3_ (5.2 ng/mL, Sigma-Aldrich)] for 5 days, then followed the miRNA transfection.

### MiRNA and SiRNA transfection

miR-148b mimic, MiRNA-148b mimic negative control (miR-148b mimic-con), MiRNA-148b inhibitor, and MiRNA-148b inhibitor negative control (miR-148b inhibitor-con) were synthesized by RiboBio Co., China. Wnt-1 siRNA was purchased from Qiagen, Germany. Transfection was performed using Lipofectamine RNAiMAX Transfection reagent (Thermo-Fisher, USA) following the manufacturer's instructions.

### Immunofluorescence staining and quantification

For proliferation assay, NSCs were cultured on polyornithine/laminin-coated 24-well plates at a density of 5 × 10^4^ cells per well in growth medium for 24 h. To label the dividing cells, 5 μM 5-Bromo-2′-deoxyuridine (BrdU, Sigma-Aldrich) was added into medium during the last 8 h of culture. Cells were fixed in 4% paraformaldehyde for 20 min, incubated with 50% Formamide for 30 min at 65°C, and washed with 1 M HCl for 10 min at 37°C, and 0.1 M borate buffer for 3 min. Non-specific binding sites were blocked with 1% bovine serum albumin (BSA) and 0.3% Triton PBS for 1 h at room temperature. Then, NSCs were incubated with rabbit anti-BrdU (1: 2,000, Abcam) overnight at 4°C. After washing with PBS, cells were incubated with FITC-conjugated secondary antibodies, and followed by 4-6-diamidino-2-phenylindole (DAPI, 1: 10,000, Abcam) incubation for 1 h at room temperature. For differentiation assay, cells were fixed and incubated with blocking buffer and incubated with mouse anti-Neuron-specific class III beta-tubulin (Tuj1, 1:200, Abcam) or rabbit anti-glial fibrillary acidic protein (GFAP, 1:200, Abcam) overnight at 4°C. The next day, cells were washed and incubated with the secondary antibodies and DAPI as the same procedures described above. Cells were counted from three wells/group. Ten view fields per well were imaged under a 40X objective lens. Data is showed as the number of marker-positive cells divided by the total number of DAPI.

To determine neurogenesis in SVZ of ischemia-affected hemisphere, paraffin sections of rat brains were used for staining the following markers, mouse anti-DCX (1:100, Santa Cruz Biotechnology), mouse anti-NeuN (1:100, Abcam), and moue anti-GFAP (1:100, Abcam). All primary antibodies were incubated at overnight at 4°C, and followed by FITC-or Cy3-conjugated secondary antibodies incubation at a concentration of 1:300. To quantify the regional differences in the number of NPCs, neurons and NSCs, we used 40X objective lens to count positive cell in five view fields per section and 1-in-5 series of sections were stained for each animal.

### Quantitative real-time PCR (qRT-PCR) analysis

Total RNA was isolated from cultured cells or tissue using the miRNeasy Mini kit (Qiagen, Germany), for formalin-fixed, paraffin-embedded tissue we used miRNeasy FFPE (Qiagen, Germany). Total 10 ng of RNA from each sample was reverse transcribed using TaqMan® MicroRNA Reverse Transcription kit (Applied Biosystems). Quantification of miRNA was performed using standard TaqMan® PCR kit protocol (Applied Biosystems). The method of 2^−ΔΔCT^ was used to calculate relative miRNAs expression levels. Each sample was tested in triplicate and at least three samples obtained from independent experiments were examined. U6 snRNA served as the internal control (Thermo-Fisher; Livak and Schmittgen, 2001).

### Animal experimental protocol

Ten weeks old male Sprague-Dawley rats weighing 180–220 g were performed MCAo. The next day, animals were randomly divided into the following three groups (*n* = 10/group): Lentivirus miR-148b inhibitor (LV-148b-inhibitor, CMV-ZsGREEN1-148b-miRNAinhibitor-PGK-puromycin, 3.0 × 10^6^ IU in 3 μL saline, Genomeditech, China), LV-GFP or saline vehicle control. Rats were anesthetized with 10% chloral hydrate, and fixed on a stereotactic frame (RWD, China). The liquid was injected into the right striatum at a rate of 0.2 μL/min via a mini-pump (WPI, USA). Behavioral tests, including modified neurological severity score (mNSS) test, foot-fault test, and adhesive patch removal test were performed on day 1, day 4, day 7, and day 14 after MCAo (Chen et al., 2001; Shen et al., 2007). On day 14, all the animals were perfused using saline, followed by 4% paraformaldehyde solution. Brains were removed and fixed in 4% formalin, dehydrated, and embedded in paraffin separately. To measure lesion volume, brain slices were stained with hematoxylin and eosin (HE), and the infarct lesion volume (mm^3^) was measured as appropriate area (mm^2^) multiply section interval thickness (2 mm).

### Behavioral tests

mNSS was measured according to the published record (Chen et al., 2001). All animals were tested the abilities of motor (score 0–6), sensory (score 0–2) balance (score 0–6), reflex and abnormal movements (score 0–4). Total maximum score is 18, higher score represents severer injury. For foot-fault test, animals were placed on horizontal ladder and trained to go cross it. When testing, total steps and the foot-faults steps for left forelimb were record separately, and the results were presented as the percentage of foot-fault steps to the total number of steps (Hernandez and Schallert, 1988). For adhesive patch removal test, adhesive paper was placed at distal-radial region on the wrist of left forelimb as tactile stimuli, and recorded the time required to remove the stickers. For each animal, three trials per testing day were performed (Shen et al., 2007).

### Western blot analysis

Cells were lysed in lysis buffer containing 1% protease and 1% phosphatase inhibitor (Cell Signaling Technology) followed by centrifuging for 15 min at 15,000 rpm, 4°C to remove cell debris. Protein concentrations were determined using BCA assay (Thermo-Fisher). For each sample, 30 μg of total protein was separated by SDS-PAGE and transferred to nitrocellulose membrane. Membrane was blocked and incubated with an appropriate primary antibody and a secondary antibody. The following antibodies were used: mouse anti-β-actin (1: 5,000, Abcam), rabbit anti-Wnt1 (1: 1,000; Abcam), rabbit anti-β-catenin (1: 1,000; Abcam), rabbit anti-Cyclin D1 (1: 1,000, Abcam), rabbit anti-PROX1 (1: 1,000, Abcam), rabbit anti-NeuroD1 (1: 1,000, Abcam), rabbit anti-DCX (1: 1,000, Abcam), and mouse anti-glial antigen 2 (NG2, 1: 1,000, Abcam). Proteins were visualized by enhanced chemiluminescence (Thermo-Fisher).

### Statistical analysis

All data are presented as mean ± SE. Significant differences between groups were analyzed using One-way ANOVA. Statistical significance was set at *p* < 0.05.

## Results

### The expression of miR-148b was significantly increased in ischemic rodent brain SVZ

**Figure 1:**
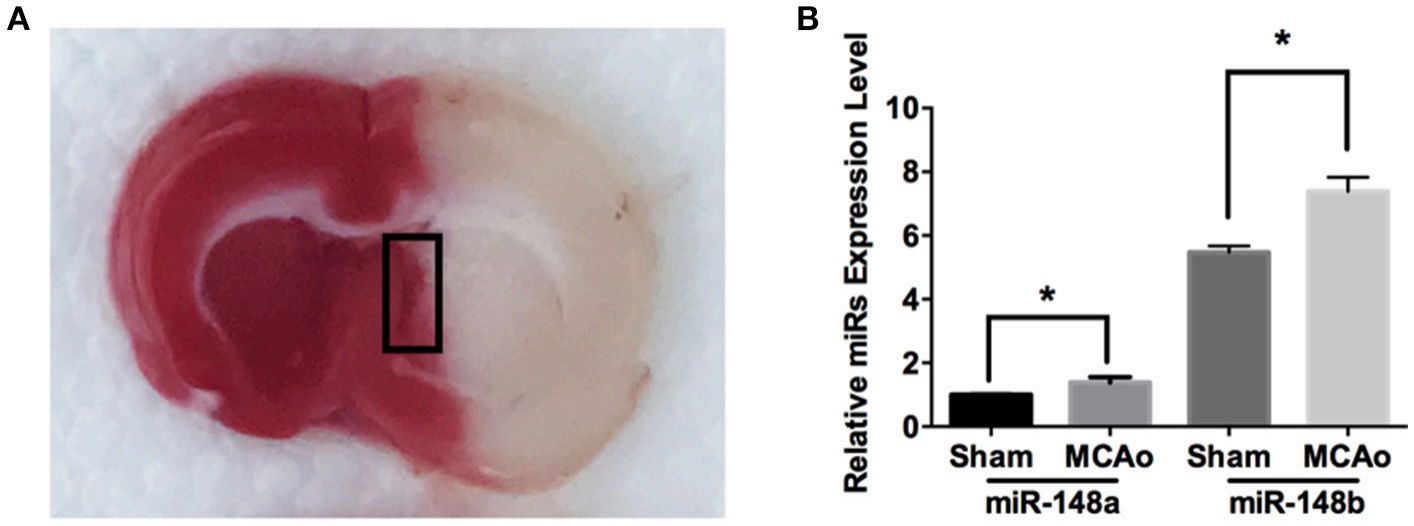
miR-148a/b expression levels changed after MCAo. **(A)** Image of TTC staining of rat brain section after MCAo. SVZ (marked with black rectangle) in ischemic hemisphere and the corresponding area from sham group rats were isolated respectively for qRT-PCR test; **(B)** miR-148a/b expression levels were measured in sham and MCAo rat SVZ area. Mean ± SE, ^*^p < 0.05, *n* = 3/group.

To explore the potential involvement of miR-148 in ischemic SVZ, sub-ventricular zone was isolated from sham-operation (Sham) and MCAo applied rat brains (Figure 1A). Quantitate RT-PCR analysis indicated the expression levels of both miR-148a and miR-148b in ischemic SVZ were obviously increased compared with sham group (Figure 1B). However, the enrichment of miR-148a in sham and ischemic brains were significantly lower than miR-148b, and the following study was focused on miR-148b.

### miR-148b directly targeted Wnt1 gene

**Figure 2:**
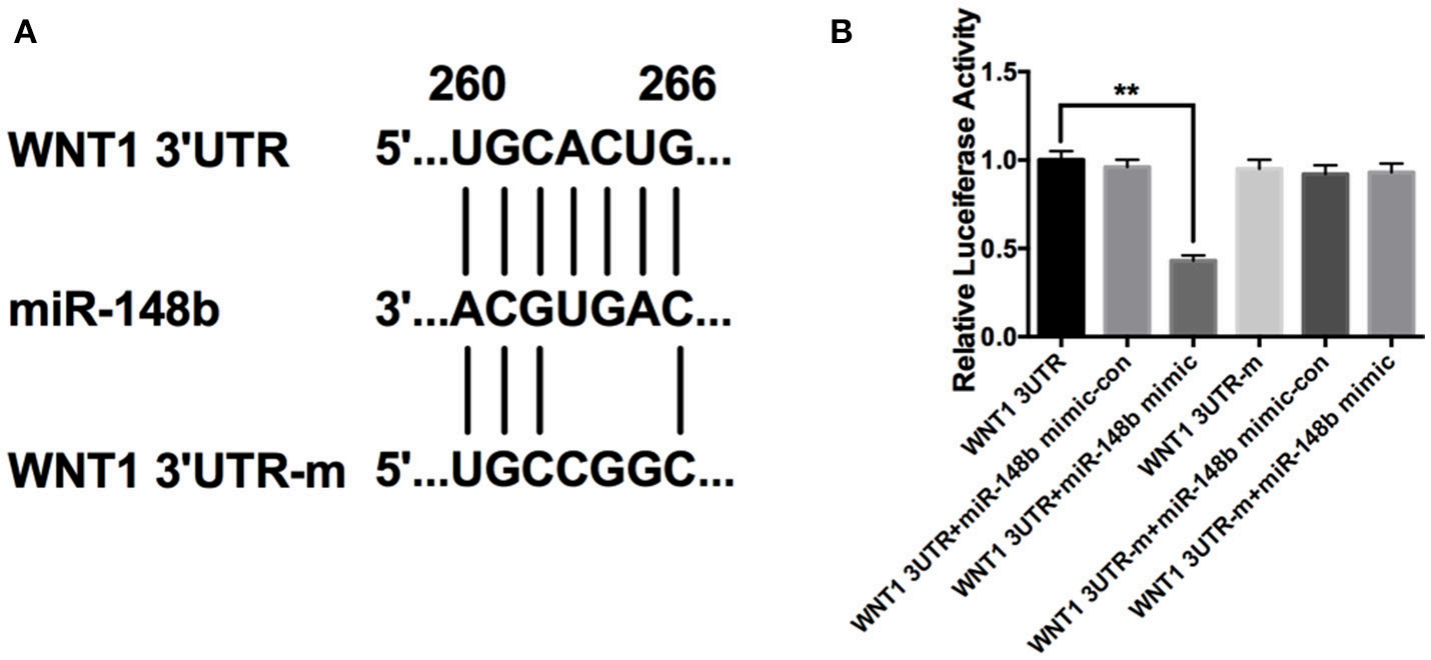
miR-148b directly targeted Wnt1 in HEK 293T cells. **(A)** Schematic diagram shows wild-type or mutated miR-148b binding site in Wnt1 3′UTR fragment; **(B)** Luciferase reporter assay data of wild type or mutant 3′ UTR of Wnt1 with miR-148b mimic-con or miR-148b mimic. mean ± SE, ^**^p < 0.01, *n* = 3/group.

Using targetscan.org, we searched predicted target genes of miR-148b. A potential miR-148b broadly conserved sequence was found to be seed matched with 3′UTR of Wnt1 mRNA (Figure 2A). To confirm the directly targeting relationship of miR-148b and Wnt1, we constructed luciferase expression vectors harboring wild type or mutant Wnt1 3′UTR binding site (WNT1-3UTR and WNT1-3UTR-m; Figure 2). Luciferase assay revealed that WNT1-3UTR had significantly lower activity than WNT1-3UTR-m in HEK-293T cells when miR-148a mimic co-transfected. In contrast, miR-148b mimics did not reduce luminescence activity when miR-148b seed sequences at 3′ UTR of Wnt1 gene was mutated (Figure 2B). These data indicated miR-148b could target Wnt1 mRNA in HEK 293T cells.

### miR-148b regulated Wnt/β-catenin signaling in SVZ NSCs

**Figure 3:**
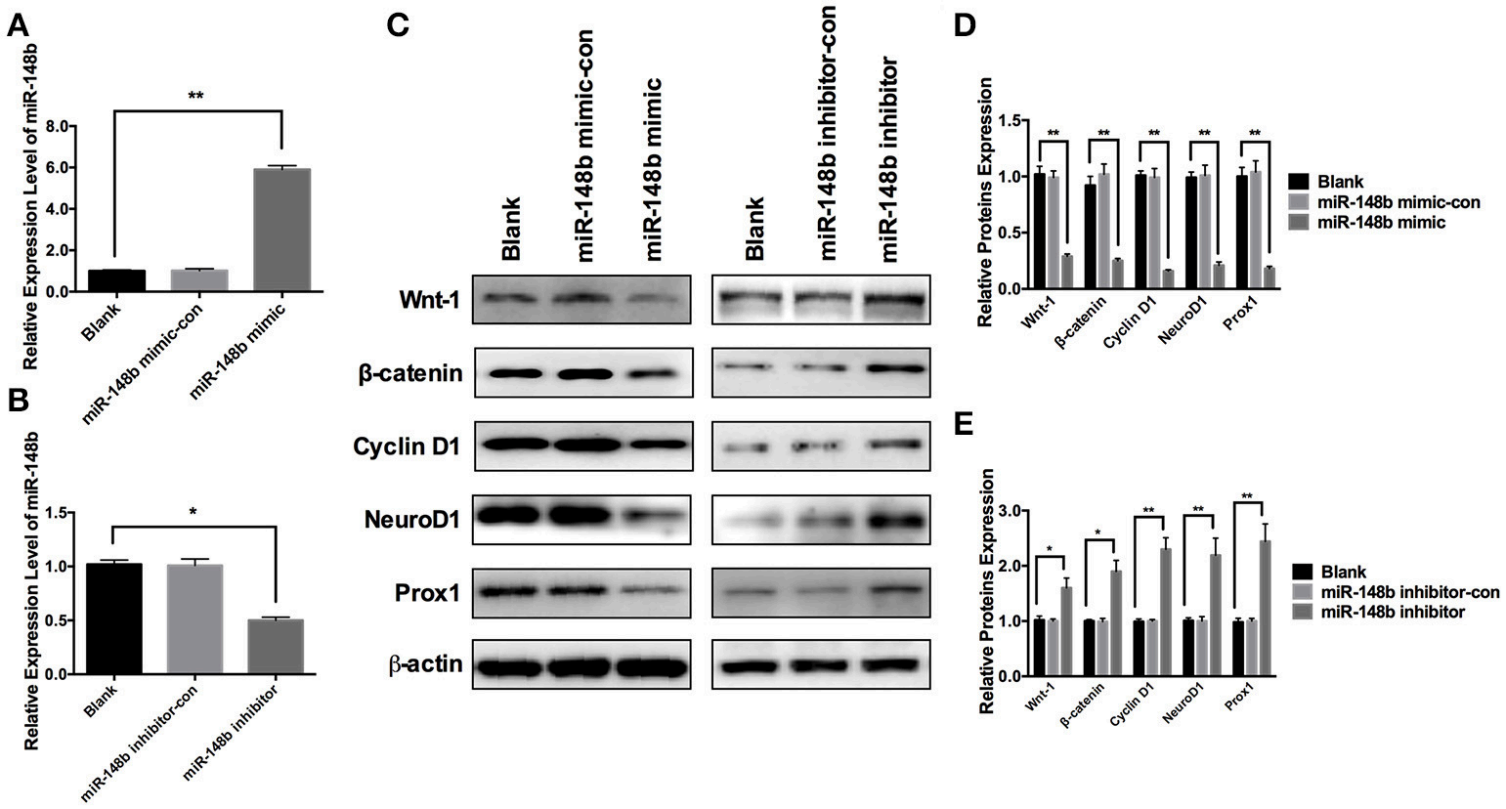
Effects of miR-148b on Wnt/β-catenin signaling activity. **(A)** miR-148b expression levels in ischemic NSCs transfected with miR-148b mimic or miR-148b-mimic-con; **(B)** miR-148b expression levels in ischemic NSCs transfected with miR-148b inhibitor or miR-148b inhibitor-con; **(C–E)**. Western blot analysis of Wnt-1, β-catenin, Cyclin D1, NeuroD1, and Prox1 expression contents and their quantitative data in ischemic NSCs transfected with miR-148b mimic, miR-148b inhibitor and their negative controls. mean ± SE, ^*^p < 0.05, ^**^p < 0.01, *n* = 3/group.

To further investigate the effect of miR-148b on Wnt/β-catenin signaling pathway, primary ischemic SVZ NSCs were transfected with miR-148b mimic and miR-148b mimic negative control (miR-148b mimic-con), respectively. The expression of miR-148b was increased by 5.92 ± 0.44 and 5.58 ± 0.38 times higher than blank (vehicle control) and miR-148b mimic-con, respectively (Figure 3A). Western blot data showed the expression of Wnt1 and β-catenin were significantly decreased (*p* < 0.05, *n* = 3) in miR-148b mimic group compared with blank and miR-148b mimic-con group. Three downstream proteins of Wnt/β-catenin pathway, Cyclin D1, NeuroD1 and Prox1 were also detected and showed significantly downregulated in miR-148b mimic transfected NSCs (*p* < 0.05, *n* = 3; Figures 3C,D). On the contrary, cells transfected with miR-148b inhibitor substantially decreased the expression of miR-148b by 0.31 ± 0.06 and 0.29 ± 0.04 times compared with blank and miR-148b inhibitor control transfected cells, respectively (Figure 3B). And the increasing proteins expression of Wnt/β-catenin signaling and downstream was also confirmed by the means of western blot (Figures 3C,E). These results further demonstrated that Wnt1/ β-catenin signaling was regulated by miR-148b in SVZ NSCs.

### miR-148b mediated the proliferation and differentiation of SVZ NSCs via Wnt/β-catenin signaling

**Figure 4:**
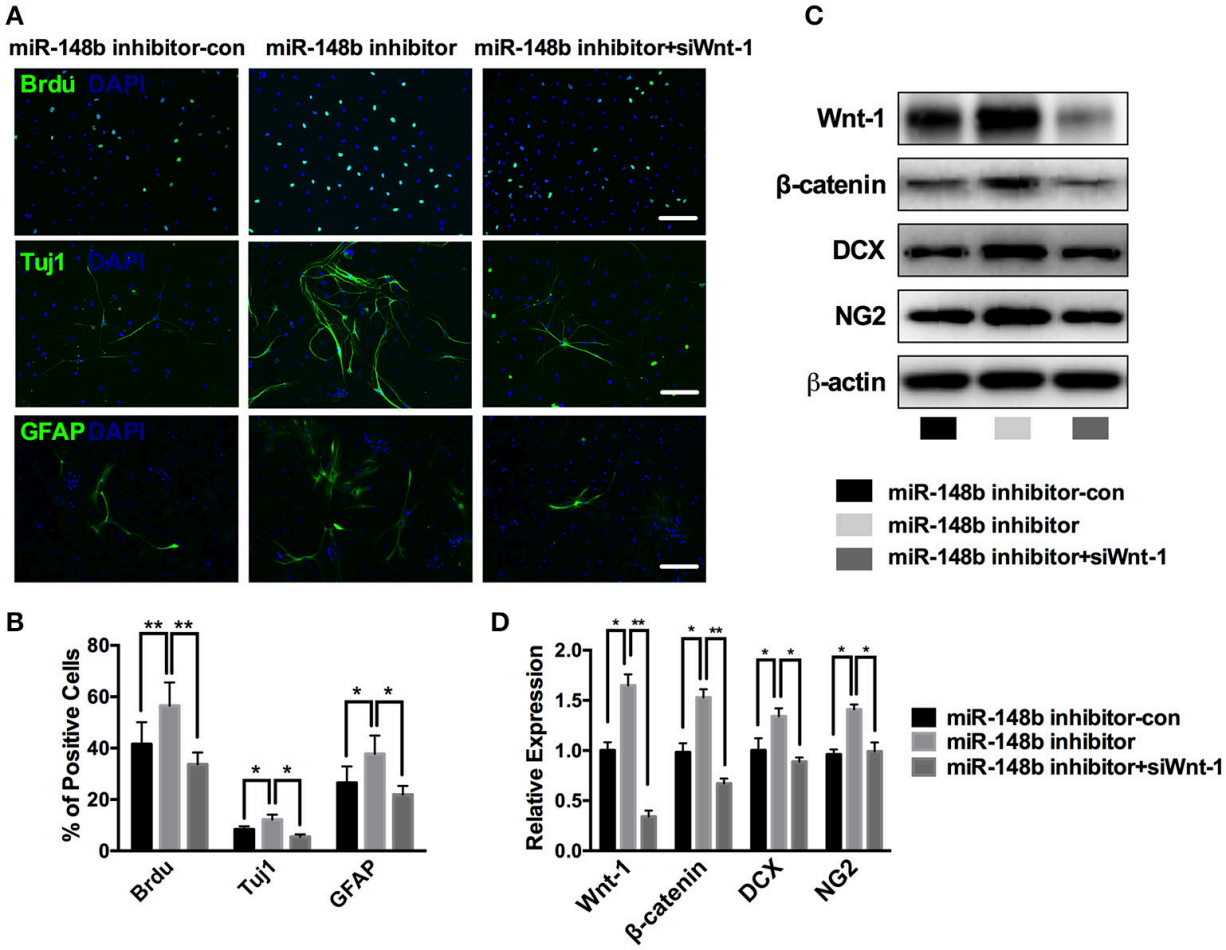
miR-148b involved in neurogenesis via regulating Wnt/β-catenin signaling activity. **(A,B)** Immunostaining images and quantitative data of BrdU, Tuj1 and GFAP positive cells in ischemic NSCs transfected with miR-148b inhibitor-con, miR-148b inhibitor, or miR-148b inhibitor with siRNA Wnt-1; **(C,D)**. Western blot analysis of Wnt-1, β-catenin, DCX and NG2 expression and their quantitative data in ischemic NSCs transfected with miR-148b inhibitor, miR-148b inhibitor-con and siRNA Wnt-1. mean ± SE, ^*^p < 0.05, ^**^p < 0.01. Scale bar = 5 μm.

The proliferation and differentiation activities of NSCs are curial for neurogenesis process. First, we tested the effect of miR-148b on SVZ NSCs proliferation. Ischemic SVZ cells cultured in growth medium were transfected with miR-148b inhibitor and miR-148b inhibitor negative control (miR-148b inhibitor-con), respectively. Immunofluorescent staining showed a robustly up-regulated BrdU-positive cell percentage as 56.5 ± 9.1% compared with miR-148b inhibitor-con group (41.6 ± 8.5%, *p* < 0.01; Figure 4A).

Then, we tested the effect of miR-148b on NSCs differentiation. Primary ischemic SVZ cells were transfected with miR-148b inhibitor/inhibitor negative control and cultured in differentiation medium for another 5 days. Immunofluorescent staining analysis revealed that suppress of miR-148b resulted in a significant increase in the number of Tuj1-positive cells (12.2 ± 1.9% in miR-148b inhibitor vs. 8.4 ± 1.2% in miR-148b inhibitor-con group, *p* < 0.05) and GFAP-positive cells (37.8 ± 3.1% in miR-148b inhibitor group vs. 26.5 ± 6.4% in miR-148b inhibitor negative control group, *p* < 0.05; Figures 4A,B). Western blot further demonstrated the expression of NG2 (neural/glial antigen 2) and DCX was also increased significantly in miR-148b inhibitor transfected NSCs than inhibitor negative control transfected cells (Figures 4C,D).

Additionally, we silenced the expression of Wnt1 in ischemic SVZ cells by Wnt1 siRNA. As shown in Figure 4C, the expression of Wnt/β-catenin signaling and the downstream proteins were obviously down-regulated when Wnt1 siRNA was applied, and miR-148b inhibitor could not increase the expression of these proteins (*p* < 0.01, vs. miR-148b inhibitor). These results indicating Wnt/β-catenin signaling mediated the neurogenesis inhibitory effect of miR-148b on SVZ NSCs.

### miR-148b inhibitor decreased the ischemic lesion volume and improved neurological recovery in MCAo rats

**Figure 5:**
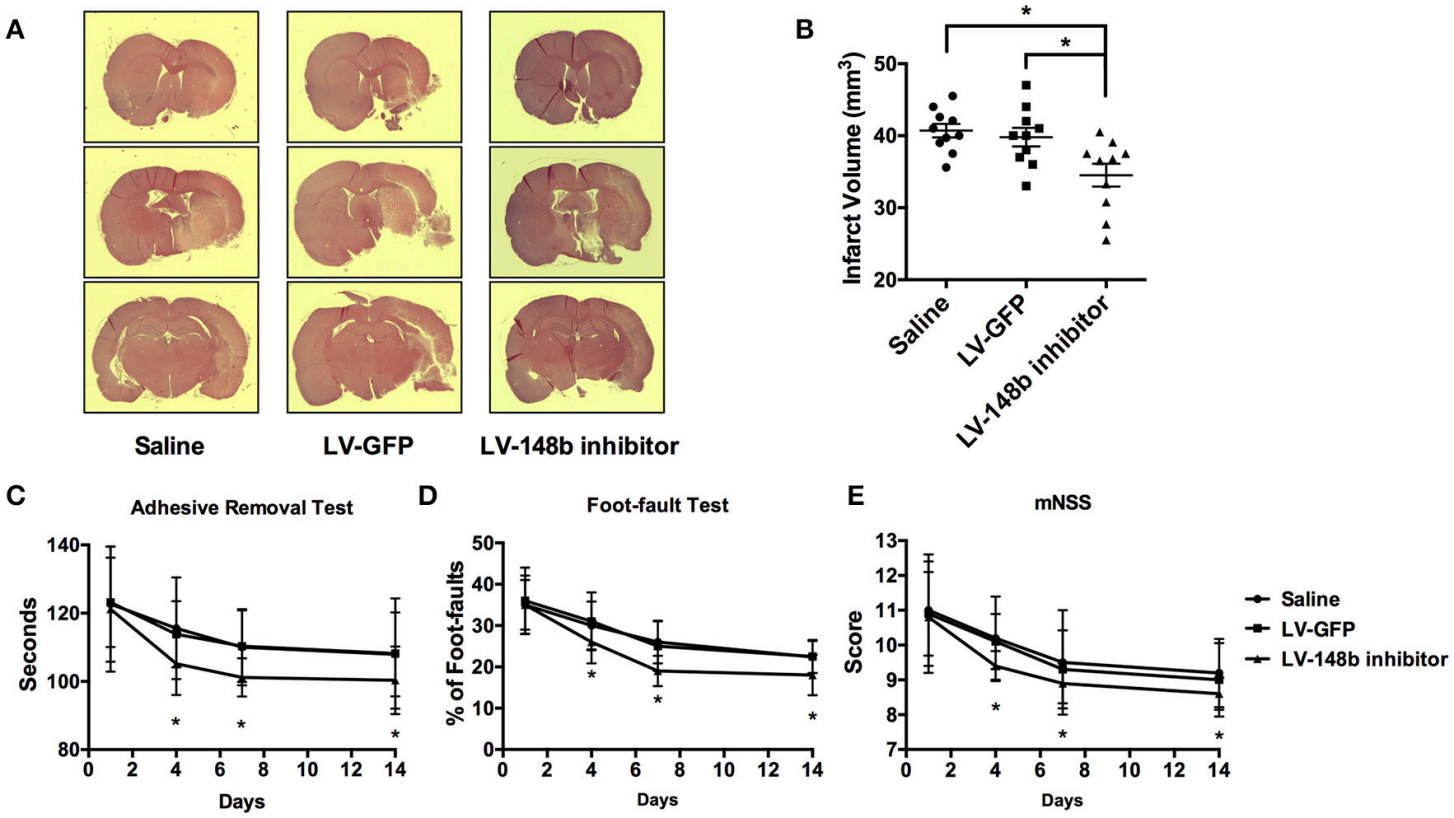
LV-148b inhibitor reduced brain infarction volume and improved neurological functional recovery after MCAo. **(A)** Effect of LV-148b inhibitor on infraction volume of rat brains after MCAo; Brain coronal sections stained with H&E show infarction from rats treated with saline, LV-GFP or LV-148b inhibitor after MCAo. **(B)** Quantitation of infarct volumes in all experimental groups. *n* = 10/group, mean ± SE, ^*^p < 0.05. **(C–E)** The adhesive-removal test, foot-fault test and mNSS scores test were performed at days1, 4, 7, and 14 after MCAo applied. *n* = 10/group, mean ± SE, ^*^p < 0.05.

**Figure 6:**
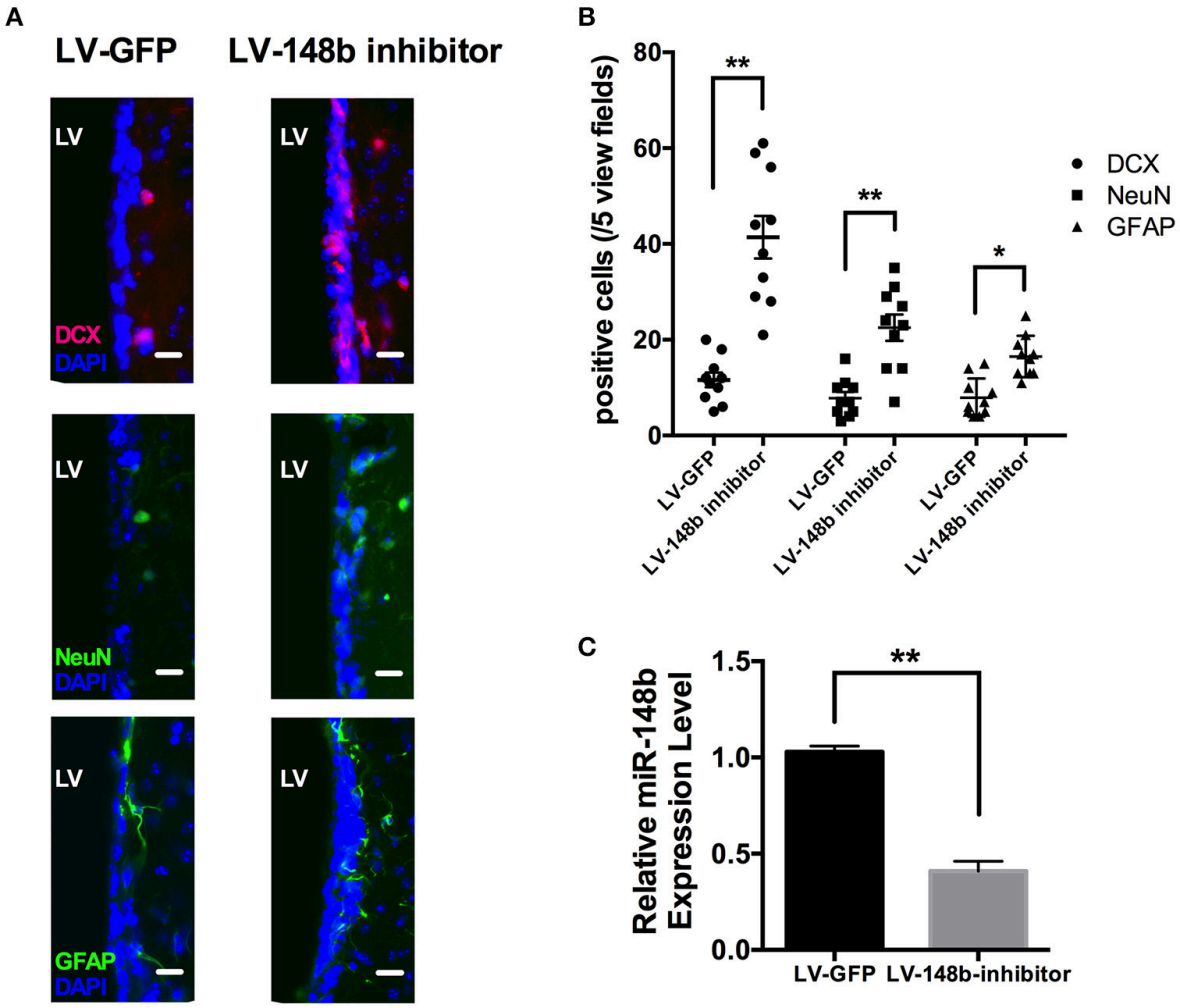
LV-148b inhibitor improved neurogenesis in rats SVZ after MCAo. **(A)** Immunofluorescence staining for DCX (red), NeuN (green), and GFAP (green) at day 14 after ischemia. Nuclei were counterstained with DAPI (Blue). Scale bar = 50 μm. **(B)** Data are represented as the number of positive cells in five view fields for each section, and three section for each animal. (*n* = 10/group. mean ± SE, ^*^p < 0.05, ^**^p < 0.01vs. LV-GFP group); **(C)** The relative miR-148b expression level in LV-148b and LV-GFP inhibitor injected rat brains SVZ (*n* = 5/group. mean ± SE, ^**^p < 0.01).

To detect the effect of miR-148b in neurological functional recovery after ischemic stroke *in vivo*, we inhibited the miR-148b expression level in rats by Lentivirus miR-148b inhibitor injection. Saline, LV-GFP, and LV-148b inhibitor was transferred to rats 1 day after MCAo was performed. All animals were scarified 14 days after MCAo. Quantitate RT-PCR demonstrated the expression level of miR-148b was significantly reduced in LV-148b inhibitor injection group (Figure **6C**), which indicating the miR-148b inhibitor transduction succeed. HE staining revealed that the brain infarct volume of rats treated with LV-148b inhibitor (28.8 ± 5.7 mm^3^) was significantly reduced than saline and LV-GFP administrated group (40.3 ± 4.5 mm^3^ and 39.2 ± 4.8 mm^3^, Figures 5A,B). Functional behavior test results demonstrated LV-148b inhibitor group showed a better neurological functional recovery outcome compared with saline and LV-GFP groups at day 4, day 7, and day 14 (Figures 5C–E). These data strongly indicated LV-148b inhibitor reduced the infarct volume and improved neurological function recovery of ischemic stroke rats.

To further explore the effects of miR-148b on neurogenesis *in vivo*, Paraffin coronal sections were used for DCX, NeuN, and GFAP staining. Compared with LV-GFP injection group, quantity of DCX^+^, ${\text{NeuN}}_{,}^{+}$ and GFAP^+^ cells in SVZ of LV-148b inhibitor treated rats were significantly higher at day 14 after MCAo (Figures 6A,B). Adult NSCs are radial, astrocytes-like GFAP^+^ cells. LV-148b inhibitor injection obviously increased the expression of GFAP, and induced the differentiation of NSCs into neuroblast which express DCX as a marker. Mature neurons in SVZ were marked with NeuN staining, according our results, LV-148b inhibitor up-regulated the number of mature neurons at day 14, indicating neurogenesis was improved in SVZ and this process could at least partially explain the effects of miR-148b inhibitor on neurological function recovery after stoke.

## Discussion

Ischemic stroke is a high mortality and disability rate disease with limited therapy (Donnan et al., 2008). Currently, tPA is an only FDA approved pharmacological agent for the treatment of acute ischemic stroke (Alvarez-Sabín et al., 2003). Recent studies indicated miRNAs profile changed in numerous central nervous system injuries including stroke, which reveals miRNAs may participate in the cellular response to ischemic damage (Ziu et al., 2014). Accumulated studies indicate regulating miRNA expression in ischemic stroke models could benefit neurological recovery outcome (Wang et al., 2015; Zhang N. et al., 2016).

The expression of miR-148/152 family could be altered by exposure to certain physical and chemical factors (Chen et al., 2013). MiR-148/152 family members are down-regulated in various cancer cell lines, which indicates the members in this family potential effect in regulating cell cycle, proliferation, and apoptosis (Yu et al., 2009; Song et al., 2011, 2012). Several studies showed miR-148/152 family members regulate not only proliferation but also differentiation process in stem cells (Yu et al., 2012; Fujiwara and Ozaki, 2016). In the present study, the expression of miR-148a and miR-148b both increased in ischemic rat brain SVZ, we speculate that reduction of blood flow and oxygen input may induce miR-148a/b expression. Due to the low enrichment of miR-148a in SVZ, we focused our study on miR-148b.

Wnt1 is a miR-148b target gene. When accumulate in cells, Wnt protein activates Wnt/β-catenin signaling pathway by directly binding to Fz receptors and promoting β-catenin accumulation and nuclear localization (Nusse, 2005). Activated Wnt/β-catenin signaling participate in neuronal recovery process by regulating the expression of neurogenesis related proteins, including Neuro D1 and Prox1 (Harland, 2001). As shown above, down-regulated miR-148b in ischemic SVZ cells enhanced expression levels of Wnt1, β-catenin, and Cyclin D1, which indicated an activation of Wnt/β-catenin pathway. Nevertheless, over-expression of miR-148b suppressed Wnt/β-catenin pathway activity. These results are consistent with the earlier demonstration that miR-148b reversely correlated with Wnt/β-catenin pathway activity (Liu et al., 2015). Neuro D1 is a transcription factor that promotes neuronal development in NSCs and Prox1 is necessary for promoting new born neuron to mature, both of them play an important role in the NSCs differentiation process (Kalani et al., 2008; Varela-Nallar and Inestrosa, 2013). Cycling D1 is a cell cycle check-point protein, which has effect on regulating cell proliferation ability (Motokura et al., 1991). According to our data, the expression of NeuroD1, Prox1, and Cyclin D1 were obviously increased after Wnt/β-catenin signaling activated by miR-148b inhibitor, which partly explained the action mechanism of miR-148b on neurogenesis.

In adult mammalian brain, SVZ serves as a reservoir of progenitors, the cell populations contained in SVZ could be used in neuroregenerative therapy (Alvarez-Buylla and Garcia-Verdugo, 2002). NSCs from adult SVZ are in distinct stages of differentiation. New born neurons (Type A cells) migrate into the olfactory bulb, then mature into local interneurons. The division of B (SVZ astrocytes) and C (highly proliferative precursors) cells suggests that one or both of these cell types could be involved in the generation of the new neurons. There is also evidence showed that SVZ cells are the primary precursors of new neurons (Doetsch et al., 1999). In the present study, ischemic NSCs with decreased miR-148b showed significantly higher percentage of BrdU^+^ cells and DCX expression level, indicating self-renewing progenitor population was raised. After EGF and FGF-2 were withdrawn, new born neurons (Tuj1^+^ cells) were increased with silenced miR-148b, meanwhile, Type B cells (GFAP^+^ cells) and OPCs marker NG_2_ expression were increased. These data indicated that miR-148b reversely correlated with proliferation and differentiation potential SVZ NSCs.

In order to demonstrate the neurogenesis regulating ability of miR-148b could affect stroke outcome, the neurological protection effect of miR-148b also be studied *in vivo*. The infarction volume of rats transfected with lentivirus miR-148b-inhibitor showed significantly reduced and the functional test results suggested miR-148b inhibitor improve the neurological functional recovery after ischemic injury. These data revealed miR-148b expression implied the recovery outcome in ischemic stroke. Neurological functional outcomes are highly correlated with the infarct volume; multiple treatments were reported to exert neuroprotective effects by promoting neural cell survival and reducing apoptotic activity via activating Wnt/β-catenin signaling (Marchetti et al., 2013; Gao et al., 2016; Li et al., 2017). Hence, we cannot exclude the possibility that anti-miR-148b could induce a neuroprotection process via Wnt/β-catenin signaling. More work is needed in the future to illustrate the effect of miR-148b in neuroprotection and neuro-restoration.

In conclusion, we reported for the first time the suppression role of miR-148b in SVZ NSCs. This study indicated a novel mechanism for the miR-148b attenuated neuroprotection by inhibiting Wnt/ β-catenin signaling, which may become a potential therapeutic option for ischemic stroke.

## Author contributions

JW made the experiments and wrote manuscript draft; TC analyzed data; GS made the experiment design and wrote the manuscript draft.

### Conflict of interest statement

The authors declare that the research was conducted in the absence of any commercial or financial relationships that could be construed as a potential conflict of interest.
